# Local knowledge, exploitation and population status of *Terminalia glabrescens* Mart. in the Northeast of Brazil

**DOI:** 10.1186/s13002-026-00871-y

**Published:** 2026-03-10

**Authors:** Clarissa Lessa Nascimento, Júlio Marcelino Monteiro, José Ribamar Sousa Júnior

**Affiliations:** Programa de Pós-Graduação em Biodiversidade e Conservação (PPGBC), Laboratório de Etnobiologia e Conservação (LECON), Universidade Federal do Piauí (UFPI), Floriano, PI Brasil

**Keywords:** Plant resource use, Ethnobotany, Woody plant, Population ecology, Uso de recursos vegetais, Etnobotânica, Planta lenhosa, Ecologia populacional

## Abstract

*Terminalia glabrescens* Mart. is a native tree widely exploited in Northeastern Brazil, particularly known for its medicinal and timber properties. Although it is frequently harvested, the consequences of extractive practices have not been assessed; therefore, this study examined the uses and influences of extractivism on the species population status. Semi-structured interviews were conducted with 86 participants, who mentioned 27 different uses across six categories: medicinal, utensils, construction, fuel, food, and fodder. Three plots of 50 × 50 m were demarcated, where individuals were identified, measured for diameter, and their height. The study indicated a preference for collecting trees of tall and intermediate size and thickness, which are considered ideal and versatile for various uses. An association was found between the forms of use and the gender of the participants, with men having more citations in all use categories, particularly in the timber context. It was observed that the population of the species did not fit the “inverted J” model, suggesting that extractivism may primarily affect trees in intermediate size classes. Although this study does not directly assess levels of exploitation, the results may be attributed mainly to timber exploitation.

## Resumo

A *Terminalia glabrescens* Mart. é uma árvore nativa amplamente explorada no Nordeste do Brasil, destacando-se especialmente por suas propriedades medicinais e madeireiras. Embora seja frequentemente coletada, as consequências das práticas extrativistas não foram avaliadas, este estudo, portanto, verificou os usos e influências do extrativismo no status populacional da espécie. Foram feitas entrevistas semiestruturadas com 86 participantes, os quais mencionaram 27 formas de uso distribuídas em seis categorias: medicinal, tecnológico, construção, combustível, alimentação e forragem. Três parcelas de 50 × 50 m foram demarcadas, onde os indivíduos foram identificados, medidos em diâmetro e tiveram a altura visualmente estimada. O estudo indicou uma preferência pela coleta de árvores de tamanho e espessura altas e intermediárias, consideradas ideais e versáteis para diversos usos. Houve associação entre as formas de uso com o gênero dos participantes, em que os homens tiveram mais citações em todas as categorias de uso, principalmente no contexto madeireiro. Observou-se que a população da espécie não se adequou ao modelo de “J invertido”, sugerindo que o extrativismo pode afetar, sobretudo, as árvores que estão nas classes intermediárias. Embora este estudo não avalie diretamente os níveis de exploração, este resultado pode ser atribuído principalmente à exploração madeireira.

## Introduction

From the understanding of plant resource use patterns and ecological analyses, it is possible to elucidate how the collection patterns of certain species can exert intense use pressure, often leading to eventual local extinction [1]. Unregulated harvesting can directly impact on the population structure and dynamics of plant species subjected to extraction, resulting in decreased growth and reproduction rates of individuals [2]. This impact of exploitation is intrinsically related to the part of the plant being harvested, collection patterns, and species regeneration potential [3].

It is important to emphasize that each use associated with a plant has unique characteristics, which may vary depending on sociocultural and environmental contexts. This is evidenced by the fact that knowledge and use of plant resources are not uniform across genders, seemingly determined by the social roles men and women play [4]. The practices of men and women related to plant use are intrinsically linked to social, cultural, and economic factors, which can influence the conservation and valuation of natural resources [5]. In general, the studies that looked at gender, especially with a focus on medicinal plants, indicated that women were able to identify more plants than men [6, 7], with gender being considered the predictor variable for the number of species known [7–9]. Or even studies looking at specific categories of use, such as timber [10] and medicinal [11], for example, or even the main categories of use in general [7]. In this context, gender functions as a key analytical category, as the social division of labor shapes distinct patterns of interaction with biodiversity [12]. Drawing on [12], understanding these gendered relations is essential for conservation, as they are associated with differentiated impacts of resource exploitation. From this perspective, the pressure on plant resources due to their different uses and availability increases the risk of exploitation occurring at detrimental levels, contributing to population decline.

Studies focusing on the use and conservation aspects of plant species often utilize combined ethnobotanical and ecological information to diagnose the population status of species facing greater use pressure [13–15]. Within this context, the *Terminalia glabrescens* Mart, popularly known as ‘catinga de porco’, emerges as an intriguing subject of study, given the medicinal significance of the genus [16], especially due to the bark extraction of *T. glabrescens* [16]. According to these authors, the extraction of the bark from the ‘catinga de porco’ tree should be done in the smallest possible size, thus contributing to its regeneration. Additionally, species of the Combretaceae family are widely distributed in ecotone areas between the Cerrado and the Semideciduous Seasonal Forest [17]. Representatives of the genus *Terminalia* are used in traditional medicine in several countries due to their rich source of secondary metabolites [18–20], which also stand out for their timber and fuel properties [21]. *Terminalia glabrescens* is a native species that has been extensively utilized by local communities in Northeastern Brazil for timber and medicinal purposes.

Although there are pharmacological records of *Terminalia glabrescens* [22, 23], there are no studies detailing the local ecological knowledge associated with the species, especially regarding use categories and the importance of this plant resource for the subsistence of local populations and biodiversity, especially considering the gender variable (men and women). Thus, this study aimed to assess whether the extraction of *Terminalia glabrescens* (forms of use, exploited parts, and collection patterns) could affect the species’ population status in an exploitation area.

This study focuses on the analysis of the population status of *Terminalia glabrescens* in the area exploited, assessing the impact of use and collection patterns on the sustainability of the species. To achieve this, we combine quantitative and qualitative ethnobotanical approaches to investigate the different uses and the main influences. The hypothesis suggests that the participants’ practices of use significantly affect the population status of *Terminalia glabrescens*, resulting in changes in the population dynamics that may lead to its decline. Additionally, the influence of gender on different uses of the species was analyzed. The hypothesis related to gender is based on the division of labor within the community. In this context, men and women perform specific tasks, and therefore we hypothesize that the impact on the population dynamics of *T. glabrescens* differs by gender, since harvesting methods (e.g., bark removal versus whole-tree felling) vary according to these socially defined roles.

## Methods

### Study area

**Fig. 1:**
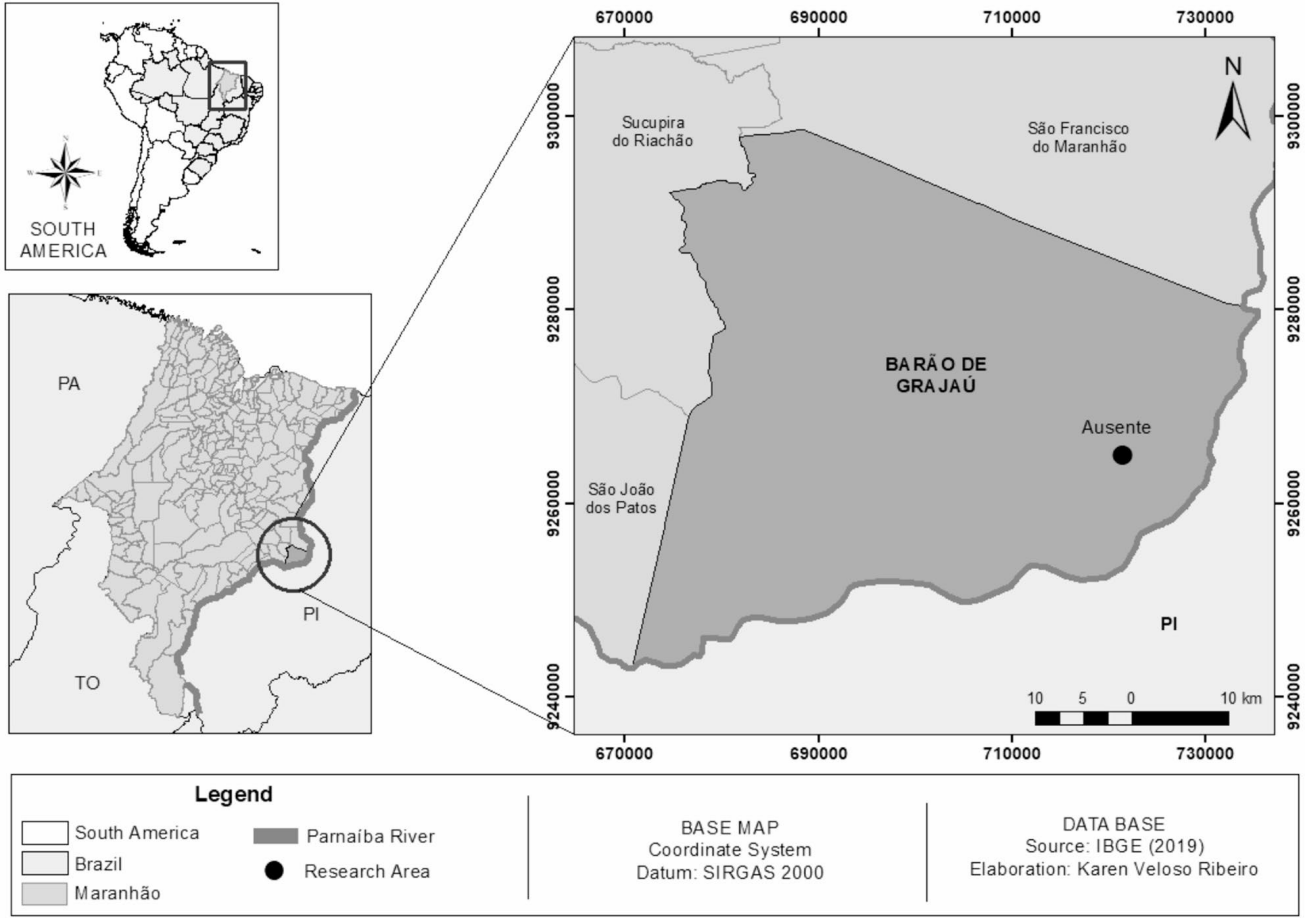
Location of the rural community of Ausente, municipality of Barão de Grajaú, state of Maranhão, Northeastern Brazil, and areas of phytosociological and ethnobotanical collections of *T. glabrescens*

This study was carried out in the rural community of Ausente, municipality of Barão de Grajaú, state of Maranhão (Fig. 1), Northeastern Brazil (6° 38’46.98” S and 42°59’49.14” W). The region has a sub-humid tropical climate with temperatures ranging from 27 °C to 37 °C. Precipitation is well-defined in summer and rare in winter, concentrated between November and April, with an increase in temperature from May to October. According to the Köppen-Geiger climate classification, the region’s climate is type ‘Aw’, which characterizes a tropical climate with a well-defined dry season during the winter period [24]. The region is characterized as an ecotone between the Caatinga and Cerrado biomes, consisting of trees and shrubs ranging from 3 to 12 m in height, structured in two strata: an arboreal/shrub stratum with sparse and twisted trees and another herbaceous/grass stratum [25]. The participants are predominantly family farmers (see Table 1) whose livelihoods are closely tied to the local environment, thereby fostering a strong dependence on natural resources.

*Terminalia glabrescens* is a tree species widely distributed throughout Brazil, occurring in the Cerrado (savanna) biome. In Maranhão, its occurrence has been recorded in riparian forests, with no records of its presence in conservation units [26]. According to these authors, the species flowers from July to October and bears fruit in October. A recent study on the regeneration of two medicinally important species showed that plant height and stem thickness did not influence bark regeneration in *T. glabrescens*, which exhibited a higher regeneration rate than the other species studied, *Copaifera langsdorffii* [17]. Based on these findings, the authors suggest that bark extraction should preferably be carried out on larger and thicker individuals. In the Ausente community, the species is distributed across native vegetation fragments surrounding the residential areas. These areas function as communal lands (common-pool resources) rather than being divided into individual private plots and are used collectively by all community members for harvesting. However, for the population structure analysis, sampling was conducted using standardized plots, as detailed in the following sections.

### Data collection

Initially, an exploratory survey was conducted to confirm the occurrence of the species in the selected locations and evidence of use. The study was approved by the Research Ethics Committee of the Federal University of Piauí (CEP/UFPI), process number 64256622.1.0000.5660, registered in the Biodiversity Authorization and Information System (SISBIO), process number 87,073, and the National System for the Management of Genetic Heritage and Associated Traditional Knowledge (SISGEN), registration - A86B9BA. Initially, we contacted the local health agent to identify potential participants who effectively manage and use the species among the community’s 150 households. Although households were used to map the community, the individual was the primary sampling unit. In cases where multiple residents in the same household were recognized as active users or knowledge holders, each was invited to participate independently. This approach ensured that the diversity of individual experiences and specialized knowledge within the community was captured, regardless of domestic organization. Subsequently, these participants acted as the starting point for the snowball sampling method [27], supported by key informants. Furthermore, the inclusion criteria adopted were: a minimum age of 18 years, use of the species, and local recognition for direct involvement in the collection or management of *Terminalia glabrescens*. Semi-structured interviews [27] were conducted, addressing the participants’ socioeconomic profile (age, gender, and occupation), the plant parts used, forms of use and preparation, the period and frequency of collection, as well as extraction patterns. Additionally, information related to the criteria for selecting plant individuals was recorded, including preferences regarding the diameter and height of the trees most frequently exploited.

A total of 86 people were interviewed, representing 53.3% of the local population, and it was observed that ages ranging from 24 to 76 years interviewees, a higher proportion of men was observed with ages ranging from 24 to 76 years and a predominance of individuals aged between 40 and 60 years. A large proportion of the interviewees were engaged in agricultural activities (28 participants), with incomes of up to one minimum wage and educational attainment corresponding to completed primary education, indicating socioeconomic challenges in the profile of the studied population, as presented in Table 1.

**Table 1:** Socioeconomic characteristics of the interviewees from the rural community of Ausente, municipality of Barão de Grajaú, state of Maranhão, Northeastern Brazil (*n* = 86)

Variable	Category	Women(*n* = 36)	Men (*n* = 50)
Age Group	< 40 years	7	9
	41–60 years	20	29
	> 60 years	9	12
Education Level	Elementary school or less*	28	42
	High School or Higher Ed.	8	8
Monthly Income	Up to 1 Minimum Wage	21	34
	Above 1 Minimum Wage	3	9
	No Income	12	7
Occupation	Rural / Construction	5	34
	Domestic / Self-employed	26	9
	Others / Unemployed	5	7

To assess the population status of *T. glabrescens*, three of 50 × 50 m plots (sampling units) were established, covering a total area of 0.75 hectares. These plots were selected in the vicinity of the community in areas where traces of timber extraction were identified, specifically signs of bark removal from the species under study. Such evidence included visible cutting marks on tree stems and areas of exposed bark, indicating collection activity. It was not possible to establish a separate control population, as the sampled areas within the accessible forest fragments exhibited some level of historical or recent harvesting. Therefore, the study focuses on a gradient of use within the exploited zones, where plant individuals are selected by local inhabitants based on factors such as stem diameter and ease of access. These traces included visible cutting marks on the trees and areas with removed bark, indicating collection activity. Individuals of *T. glabrescens* were classified into height and diameter classes as follows: individuals with diameter at breast height (DBH) > 30 cm were considered trees, those with DBH between 11 and 30 cm were classified as juveniles or intermediate, and individuals with DBH < 10 cm were considered seedlings. In all plots, individuals were tagged with aluminum plates, each with a unique number for tracking, and were measured for height (in meters) and circumference (in centimeters) to assess the impact of the collection on the trees. Individual height measurements were estimated visually, as this represents a viable and cost-effective approach supported by literature for natural forest inventories [28], while circumference measurements were taken with a measuring tape. Individuals of *T. glabrescens* were further grouped into seven DBH classes: A: 3–6 cm; B: 6–9 cm; C: 9–12 cm; D: 12–15 cm; E: 15–18 cm; F: 30–33 cm; G: 45–48 cm. For height, individuals were classified into the following classes: 2–4 m, 4–6 m, 6–8 m, and 8–10 m, to generate demographic profiles, following [29]. For inclusion of individuals in the sample, we established the criterion of measuring the Circumference at Breast Height (CBH) from ≥ 10 cm at a height of 1.30 m above ground level [30]. For analysis, the CBH was converted to Diameter at Breast Height (DBH) based on the following formula: [DBH = (CBH / π)] [31].

### Ethnobotanical data

The aforementioned uses were organized into categories according to the citations provided by the participants, carefully considering the specific information collected during the interviews. These categories were developed and adjusted based on the relevance and frequency of the responses, ensuring they accurately reflected the local knowledge and practices [32], medicinal, aimed at the treatment of illnesses; energy, intended for firewood or charcoal production; utensils, related to the manufacture of wooden handicrafts or household utensils; construction, associated with the construction of residential structures and the building of fences; food, referring to the use of bark as a coloring agent for beverages; and forage, corresponding to the use of leaves in the feeding of cattle and goats.

To quantify agreement among informants regarding plant parts used, the Plant Part Value (PPV) index was calculated using the following formula:

$$\:PPV=\frac{{n}_{p}}{N}$$

where *nₚ* is the number of informants who cited a given plant part (e.g., bark) and *N* is the total number of informants.The Use Diversity Value (UDV) was used to assess the importance of different use categories and their contribution to the total use value of the species. This index was calculated as:

$$\:UDV=\frac{{n}_{c}}{N}$$

where *n* represents the number of use categories cited and *N* the total number of informants [33, 34].

The Use Value (UV) was calculated to determine the relative importance of *T. glabrescens* to the participants, following the formula [35]. This index was calculated at the individual level to enable statistical comparison between groups. Subsequently, differences in use values assigned by different genders (men and women) were compared using the Kruskal-Wallis test. The Chi-square test was used to check differences in use categories between men and women. Responses related to selection patterns were analyzed based on the percentage of participant preferences for the most frequently extracted tree diameter (thickness) and height classes. For analytical purposes, these characteristics were organized into qualitative classes, defined as thin, intermediate, and thick for trunk diameter, and low, intermediate, and tall for tree height. The responses were then categorized according to these classes, and the frequency of citation of each category was quantified and expressed as a proportion of the total responses.

### Demographic analysis of *T. glabrescens*

To assess the population status of *Terminalia glabrescens* in the three sampled plots, individuals were classified into height classes with 1 m intervals and diameter classes with 5 cm intervals. Subsequently, the population’s fit to the negative exponential reverse J-shaped model, characteristic of stable and self-replacing populations [36, 37], was verified. The inverted J model, also known as the inverted J distribution, describes a typical population structure in ecology, where the density of individuals is higher in smaller size classes (such as young individuals or sprouts) and decreases as one moves to larger size classes, resulting in a graphical pattern resembling an inverted letter “J” [38]. This pattern indicates a population that is in equilibrium, with a high recruitment rate of young individuals, suggesting that the species has a good regeneration potential and remains stable over time. All histograms and statistical tests were performed using RStudio software version 4.3.2 [39].

## Results

### Uses, extracted plant parts, and collection patterns

Participants mentioned 27 different uses distributed into six categories, with the medicinal category (11) being the most prominent, consisting mainly of the use of bark and leaves for treating inflammatory and gastrointestinal conditions. This was followed by utensils (7) encompassing tools, implements, and other items for domestic use made from the trunk, construction (5), fuel (2), food (1), and fodder (1) (Table 2). This diverse range of applications, particularly the dual importance for both medicinal and structural purposes (timber and utensils), reflects the species’ multi-purpose role within the community. Of the total respondents, 84 (98%) recognized *T. glabrescens* as a medicinal resource and attributed 11 therapeutic indications to it (Table 2). Medicinal use for stomach ache received the highest number of mentions (61 citations), followed by general inflammations (59 citations), with citations of use from both bark and leaves. The use of *T. glabrescens* for fencing (31), charcoal (21), and rafters for house roofs (19) also stood out, revealing the importance of the species for treating ailments and providing wood for construction and fuel (Table 2).

**Table 2:** Different uses of *Terminalia glabrescens* Mart., cited by the rural community of Ausente, municipality of Barão de Grajaú, state of Maranhão, Northeastern Brazil. NC= Number of citations. Total participants = 86

Category	Uses	Part used	NC
Food	Beverage coloring	Bark	3
Fuel	Coal	Stem (trunk)	21
	Firewood	Stem (trunk)	6
	Rafter	Stem (trunk)	19
Construction	Gate	Stem (trunk)	1
	Fence	Stem (trunk)	31
	Clapboard	Stem (trunk)	3
	Board	Stem (trunk)	4
Fodder	Feeding cattle/goats	Leaves	2
Medicinal	Cold	Leaves	1
	Diabetes	Bark	1
	Stomach ache	Leaves	8
		Bark	53
	Kidney pain	Bark	1
	Migraine	Bark	6
	Liver	Bark	7
	Flu	Bark	1
	Inflammations	Bark	47
		Leaves	11
		Root	1
	Cleans the blood	Bark	1
	Prostate	Bark	1
	Ulcer	Bark	1
Utensils	Scythe handle	Stem (trunk)	2
	Hoe handle	Stem (trunk)	4
	Chair	Stem (trunk)	6
	Door box	Stem (trunk)	2
	Flowerbed	Stem (trunk)	3
	Home shelf	Stem (trunk)	1
	Slingshot	Stem (trunk)	1
Total	27		

**Fig. 2:**
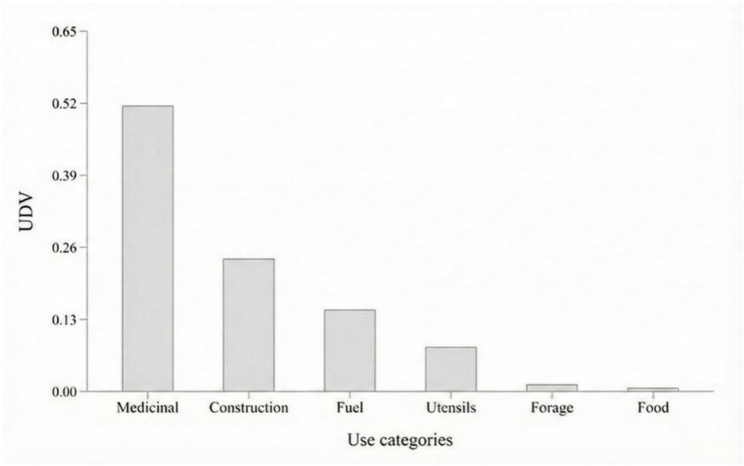
Use-Diversity Value (UDV) index for *Terminalia glabrescens* in the rural community of Ausente, municipality of Barão de Grajaú, state of Maranhão, Northeastern Brazil

**Fig. 3:**
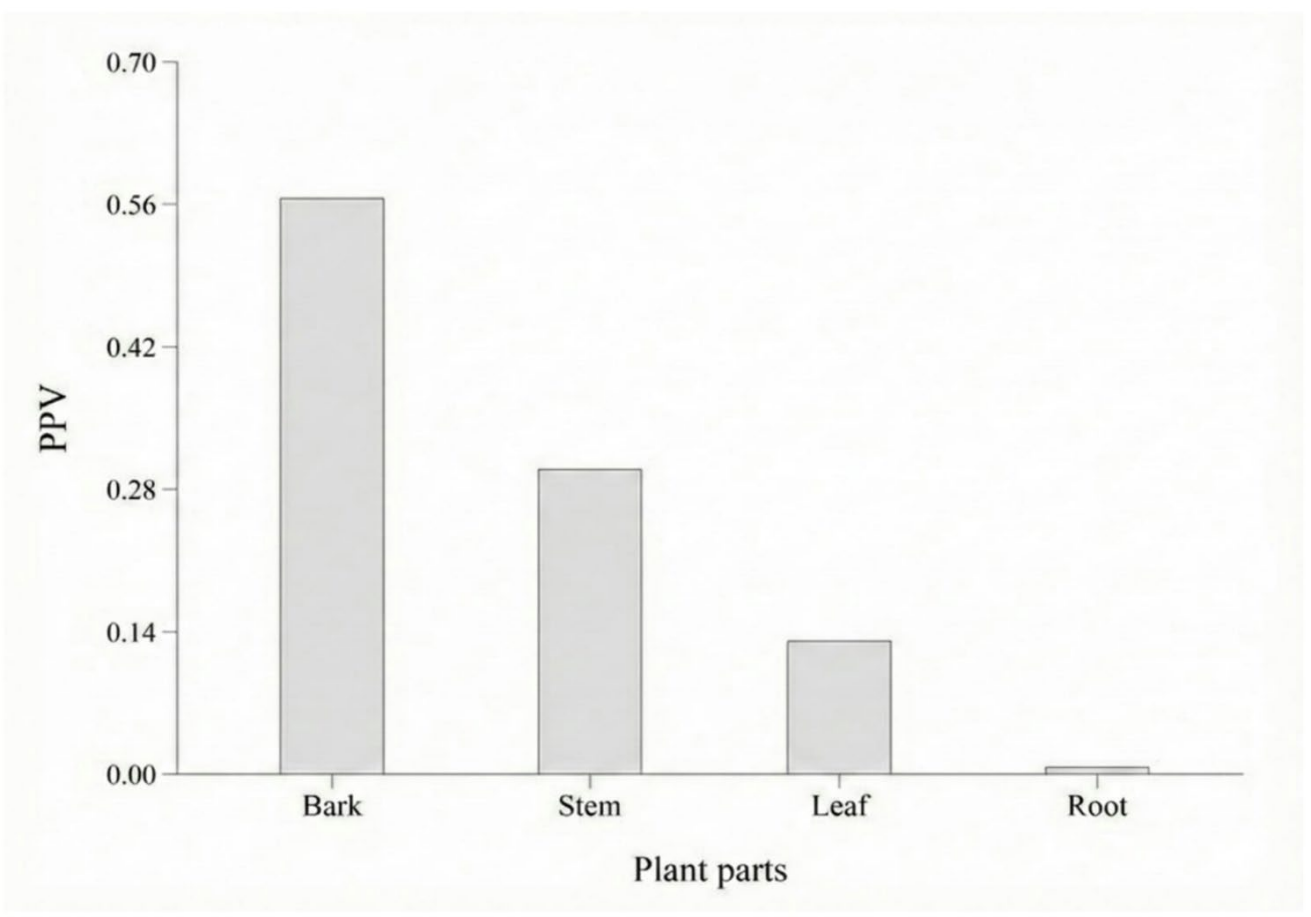
Plant Part Value (PPV) for *Terminalia glabrescens* in the rural Community of Ausente, municipality of Barão de Grajaú, state of Maranhão, Northeastern Brazil

The prominence of the medicinal category compared to others uses was evident, as it obtained the highest UDV, followed by construction, fuel, utensils, fodder, and food (Fig. 2). Regarding the plant part used as a resource, participants mentioned different uses for almost all parts, with the bark having the highest PPV, followed by the stem, leaf, and root (Fig. 3).

Regarding collection patterns, there was a preference for taller and thicker trees, as confirmed by 55% (*n* = 47) of participants expressing a preference for trees of these patterns, considered ideal and versatile for various uses within the community. The findings also revealed a preference for trees of intermediate size and thickness, representing 20% (*n* = 17) of responses, and 26% (*n* = 22) of participants indicated a preference for both sizes and thicknesses, which is generally used in the construction of fences, rafters, and tool making.

### Influence of gender on different uses of the species

**Fig. 4:**
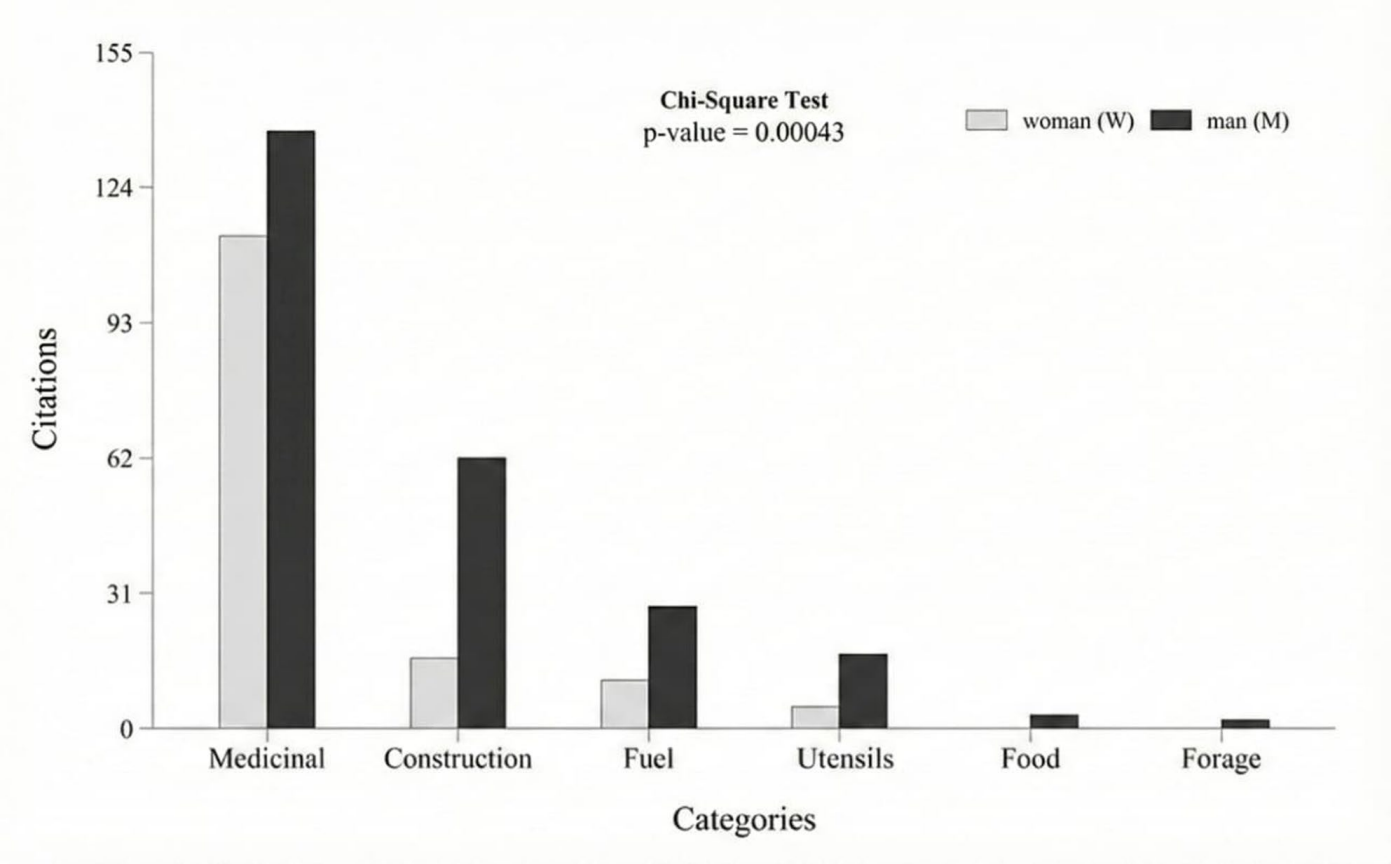
Result of the chi-square test of differences in use citations by categories according to woman (W) and man (M) for *Terminalia glabrescens* Mart. in the rural community of Ausente, municipality of Barão de Grajaú, state of Maranhão, Northeastern Brazil

**Fig. 5:**
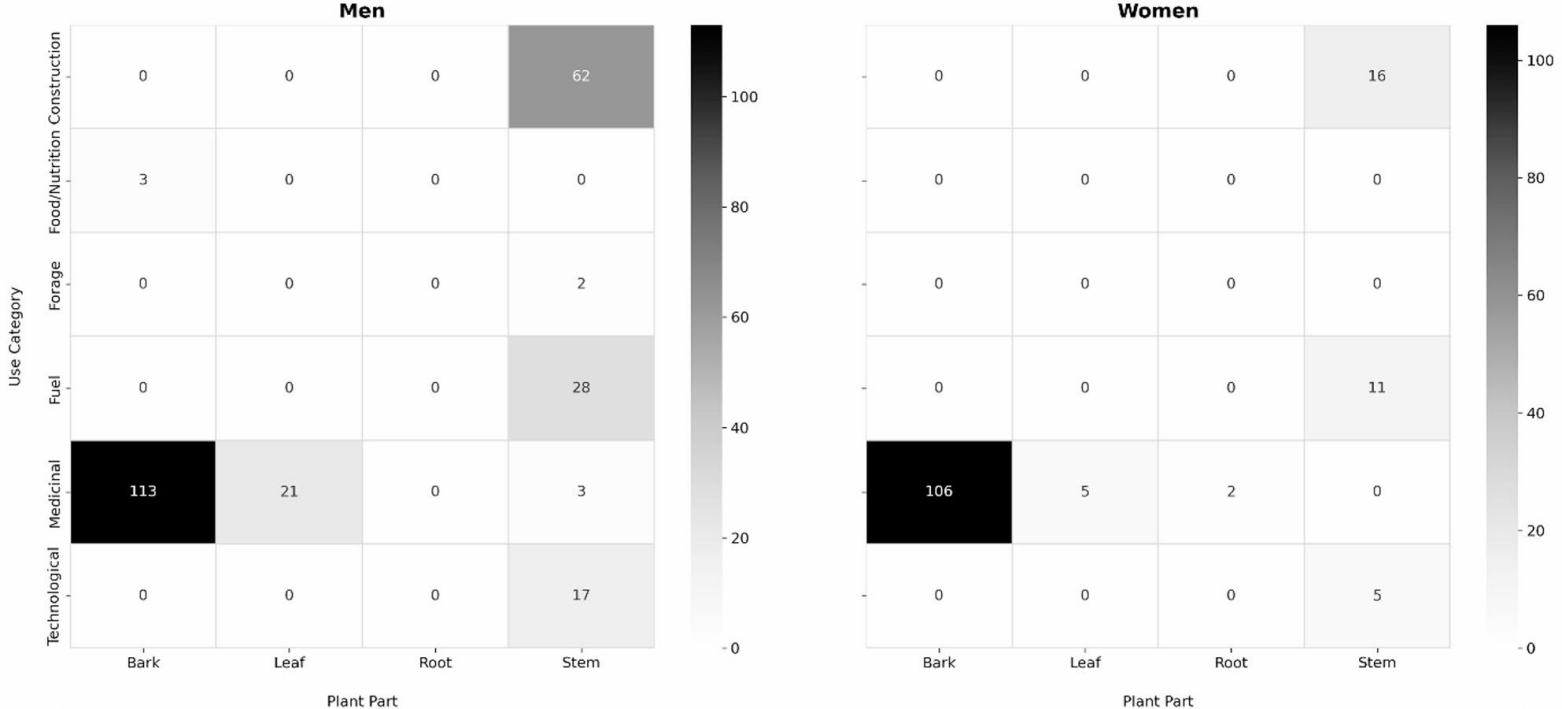
Distribution of plant uses by use category and plant part, according to gender (men and women)

The comparison between genders revealed a significant difference (H = 6.994, p-value = 0.008), indicating a positive association in preferences or use patterns of the species between genders in the studied sample. Furthermore, considering the use categories, a significant difference was also identified (X² = 22.5, p-value = 0.0004), in which men demonstrated a significantly higher number of citations in all use categories, with emphasis on categories related to construction, fuel, and utensils (Fig. 4), as well as in the distribution of plant uses by use category and plant part, according to gender (Fig. 5), in which medicinal uses are predominantly associated with bark and leaves for both genders, while uses for construction, fuel, and technological purposes are mainly related to the stem and are cited more frequently by men.

### Population Status

**Fig. 6:**
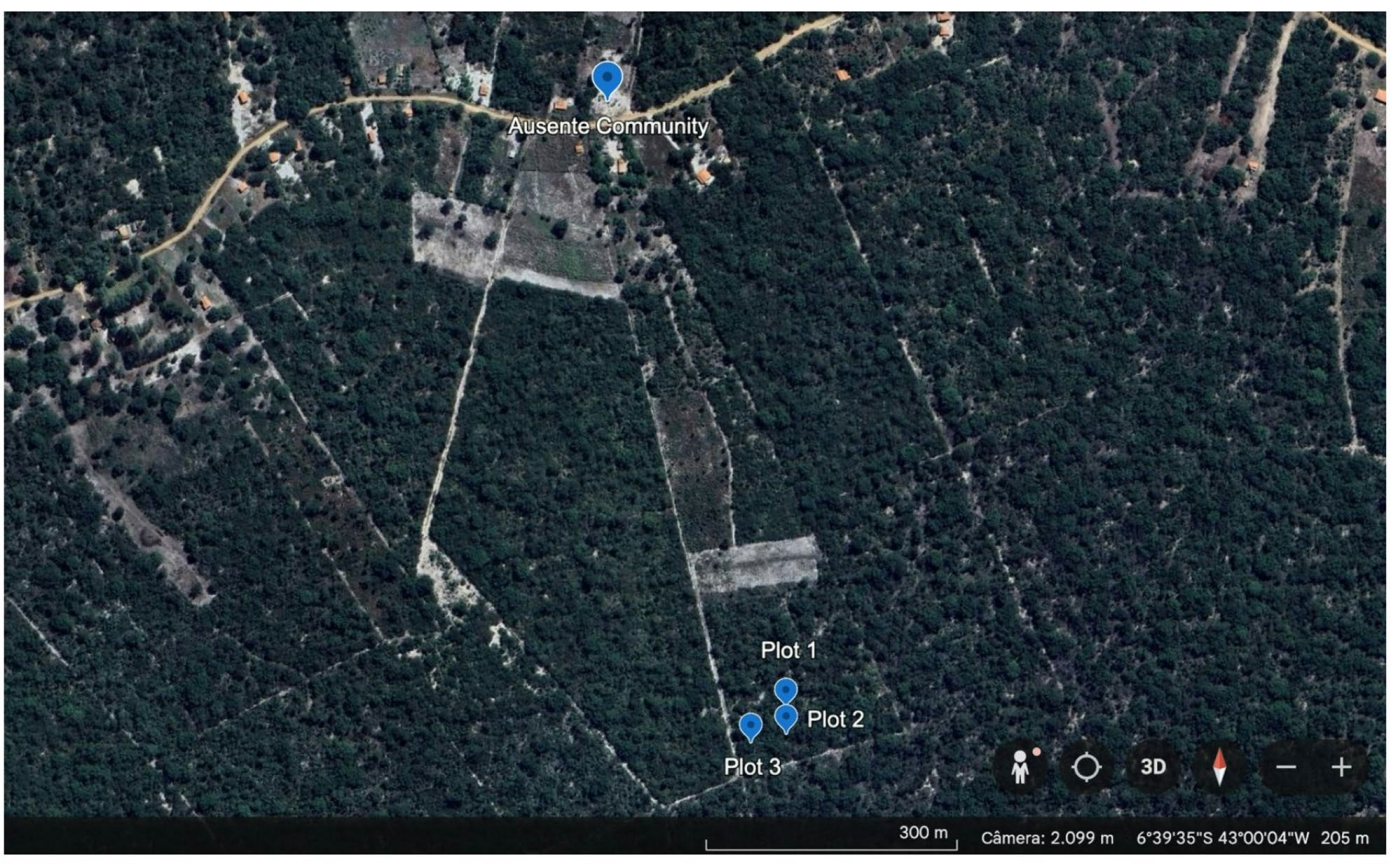
Location of the rural community of Ausente, municipality of Barão de Grajaú, state of Maranhão, Northeastern Brazil and spatial distribution of sampling units (Plot 1, Plot 2, and Plot 3) of phytosociological collections of *T. glabrescens*

A total of 34 individuals of *Terminalia glabrescens* was recorded across the three sampled plots, corresponding to a density of approximately 45.3 individuals ha⁻¹ within the total evaluated area of 0.75 ha. Most individuals were concentrated in the smallest diameter classes (3–20 cm), with a marked reduction in intermediate (20–35 cm) and larger diameter classes (35–50 cm). Regarding height, the population showed a lower frequency of individuals shorter than 2 m and a pronounced decline in the taller classes (6–10 m). Diameter (DBH) ranged from 3.18 to 46.14 cm, with a mean of 10.28 ± 10.01 cm, while tree height ranged from 2 to 10 m, with a mean of 3.90 ± 1.96 m.

**Fig. 7:**
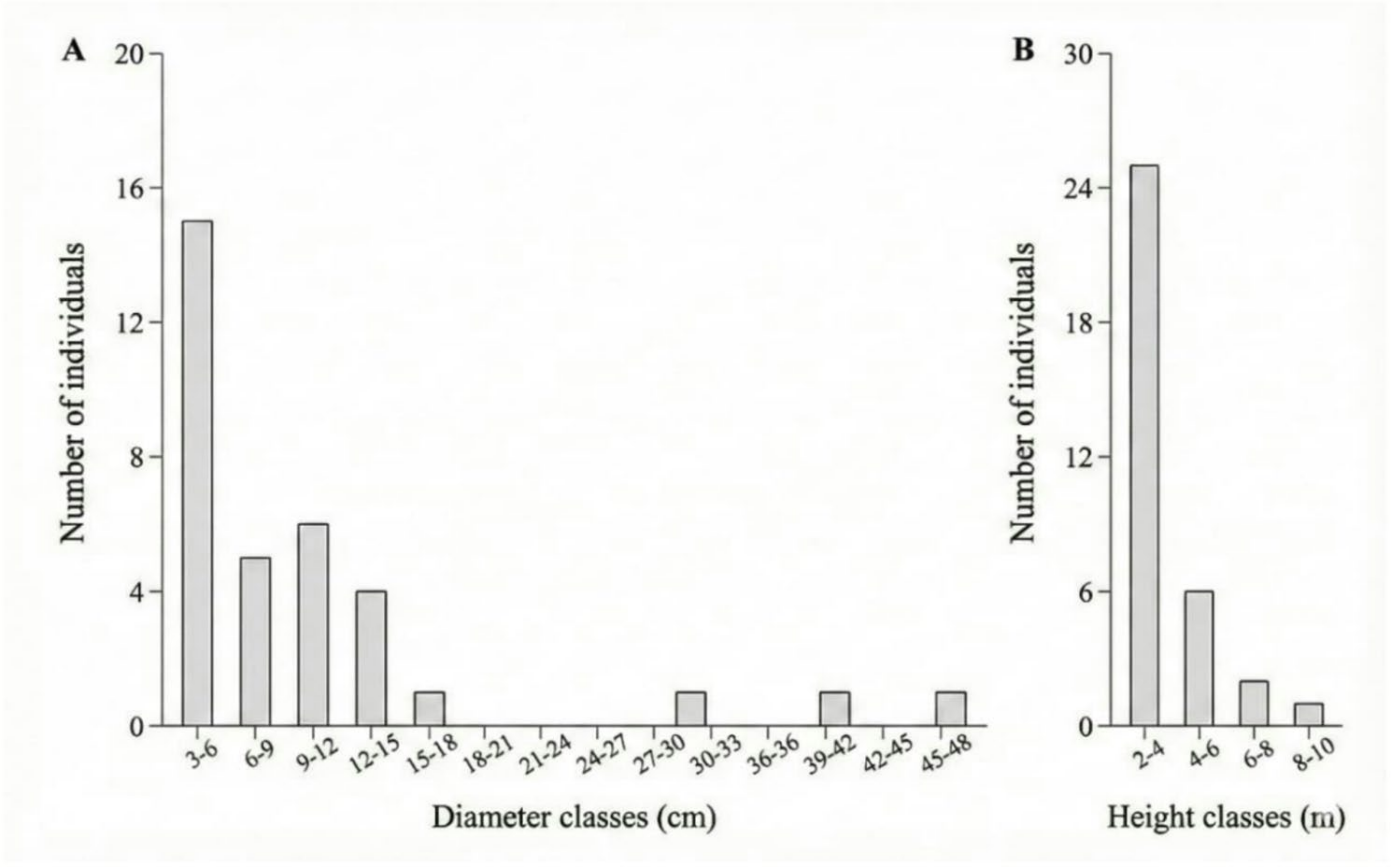
Diametric and height distribution for *Terminalia glabrescens* Mart. in the rural community of Ausente, municipality of Barão de Grajaú, state of Maranhão, Northeastern Brazil

**Fig. 8:**
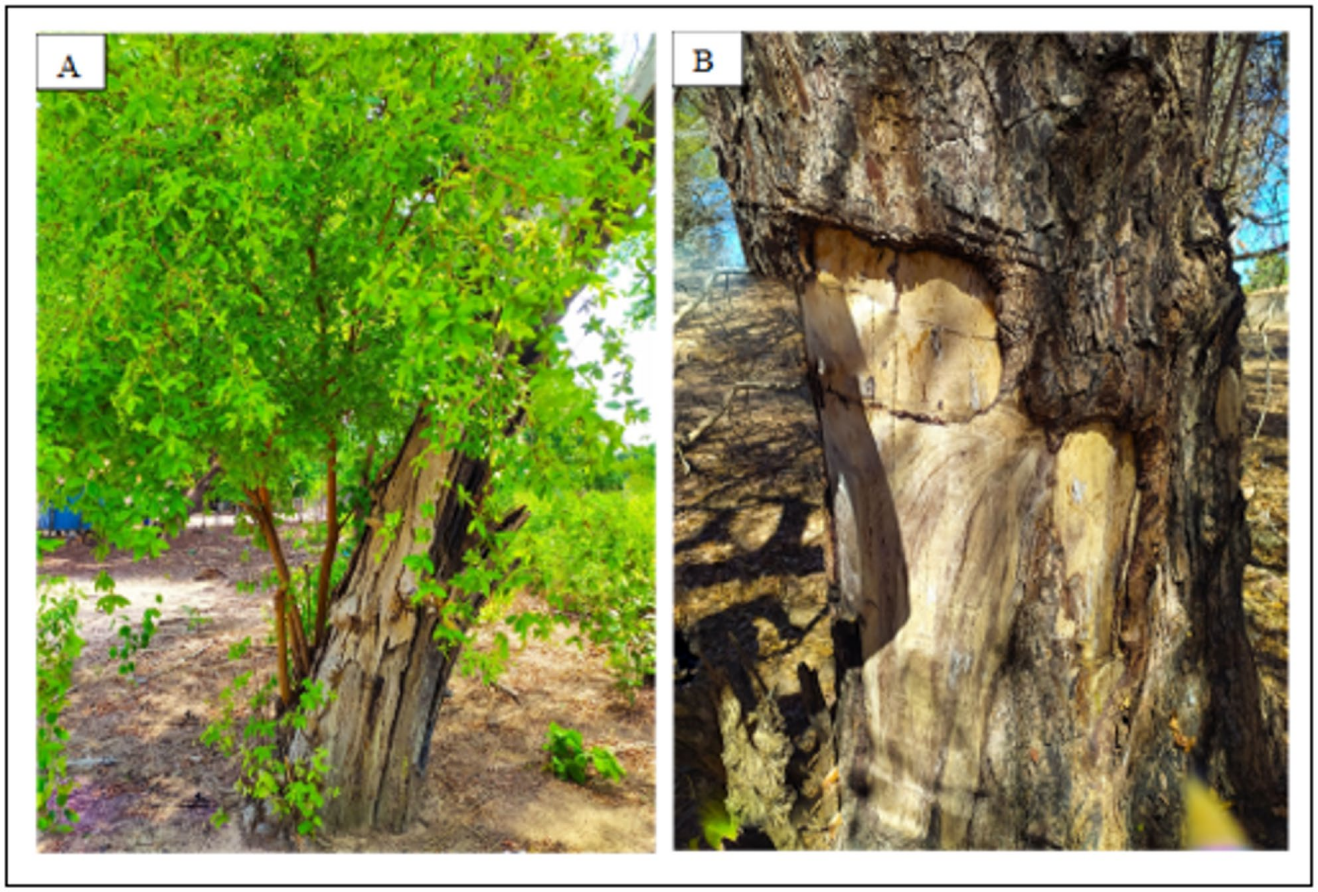
**A** Young tree growing on a dead trunk with evidence of the effects of a previous bark extraction process. **B** Evidence on the tree trunk after removing the bark of *Terminalia glabrescens* Mart. (Combretaceae) in the rural community of Ausente, municipality of Barão de Grajaú, state of Maranhão, Northeastern Brazil

Overall, Fig. 6 reveals an uneven size-class distribution, with a strong concentration of individuals in a single diameter and height class and a low number of individuals in the remaining classes. This structure deviates from the expected negative exponential pattern of a stable reverse J-shaped distribution. Although recruitment is evident through the presence of smaller individuals, the sharp reduction in intermediate and larger size classes suggests limited progression to later life stages. This pattern is likely associated with selective extraction of larger individuals, particularly for timber and construction purposes, as also indicated by the reported use and extraction patterns, as can be seen in Figs. 7 and 8.

## Discussion

### Uses, extracted parts, and collection patterns of *Terminalia glabrescens*

*Terminalia glabrescens* is important for local subsistence and economy, as residents use the species for numerous purposes, ranging from obtaining timber resources to serving as a source of fuel and medicine. This diversity of use not only demonstrates that the species is widely used but also highlights its relevance in the daily practices and traditions of the local community, representing an important resource across multiple aspects. Despite this, no statistical analysis differentiation was made regarding the different types of use, since, given the characteristics of the plant, medicinal and especially timber uses were the predominant ones. However, information related to the latter was largely not explicitly stated during the interviews (due to concerns about the legal implications of timber use) but was subtly acknowledged through expressions such as “I built my fence with it”.

The or antibacterial, antifungal, anticancer, hepatoprotective, and anti-inflammatory activities [23, 24, 40], which may be associated with the presence of pharmacological compounds such as terpenes, tannins, phenolic substances, flavonoids, lignans, and simple phenols, among others, thereby confirming the phytochemical and therapeutic properties of [21, 23, 41]. The relationship between these reported medicinal uses and the plant’s characteristics can be explained by the ontogenetic distribution of secondary metabolites. In *Terminalia* species, larger and thicker trees, preferred by the community, often possess a more developed rhytidome (outer bark), which is a primary accumulation site for tannins and phenolic compounds [21, 23]. These compounds are responsible for the antibacterial and anti-inflammatory activities cited by informants. Thus, according to the interviews, the preference for specific parts, such as the bark, is consistent with the concentration of these pharmacological agents in perennial tissues, ensuring the availability of the resource throughout the year, compared to leaves.

Although there are no studies specifically on the bark for medicinal purposes, species of the *Terminalia* genus have had compounds isolated and have been the subject of research on their phytochemical properties. A previous study found gastroprotective properties against gastric ulcers in the bark of *T. arjuna* [42]. According to [43], arjunolic acid and triterpene extracted from the bark of *T. arjuna* provided significant cardiac protection by increasing antioxidant levels, such as superoxide dismutase, catalase, and ascorbic acid.

Ethnobotanical studies on the use of stem bark for medicinal purposes report that these plant parts are mainly removed because they are more accessible and available in large-sized species and can be abundantly harvested [44, 45]. Furthermore, the preference for the bark is consistent with the concentration of these pharmacological agents in perennial tissues, ensuring that the therapeutic properties are available year-round, unlike deciduous and ephemeral parts such as leaves [33, 46, 47]. However, high levels of bark extraction can negatively affect the growth of stripped trees [48].

The use of timber was mainly cited as a source of fuel, commonly destined for charcoal and firewood production. This practice is consistent with studies indicating that the species has physical and chemical properties related to high energy performance [49, 50]. Additionally, residents stated that the wood is highly resistant to termites (Order Isoptera, Phylum Arthropoda), a fact reported in studies of species of the *Terminalia* genus, such as *T. fagifolia* Mart., which has natural resistance to decomposition caused by fungi [51].

Recent studies emphasize the importance of timber resources for rural communities [52, 53], as they are distant from urban centers and close to natural vegetation areas from which wood is frequently collected [54], not only for obtaining firewood but also for use in construction, such as fences, roofs, and boards [55], contributing to increased pressure of use on firewood resources [56].

In the community under study, where access to education and economic opportunities is limited, *Terminalia glabrescens* wood can be an accessible resource for both fuel and construction. A recent study found correlations between wood consumption and socioeconomic factors, such as income, educational level, and number of inhabitants per residence, with lower monthly income and lower educational levels being associated with greater dependence on timber resources [57], thus demonstrating the relevance of socioeconomic variables, although in the present study the focus was on the gender variable.

In the study area, we found that the extraction of *Terminalia glabrescens* trunks follows a pattern in which community members prefer to collect intermediate-sized trunks with greater diameter and height. The PPV of the bark and stem (trunk) in the community indicates that these parts are the most exploited of the species. This is evidenced by the fact that for medicinal use, height and diameter patterns may be more closely associated with the quantity of resources to be extracted than its chemical property [58]. According to the same authors [58] reported that, for trees chosen for medicinal use, the major bark extraction areas occurred in diameter classes from small to intermediate.

The preference for harvesting bark from larger individuals, as reported in the interviews, is directly supported by the population structure observed in the plots. Although the species is present in the area, the population is heavily skewed toward smaller diameter classes. The ‘thick’ trees preferred by the community represent only a small fraction of the sampled population, yet they are the primary targets for medicinal bark extraction. This selective pressure explains the uneven size-class distribution and the sharp reduction in intermediate and larger classes, as the most developed individuals are prioritized for both medicinal use and timber. As reported previously, tannin concentration was not related to species selection for medicinal use, evidencing that use preference is not determined by the chemical composition of the species but by tree diameter classes [59]. It is worth noting that excessive bark removal can be detrimental, as these trees tend to have lower survival and re-growth rates [60]. Additionally, in their study on bark regeneration in the case of *T. glabrescens* [16], indicated that it is important to harvest from large (adult) individuals, removing the bark in thin, long strips and waiting two years before harvesting from the same individual again, thus contributing to mitigate the extraction damage with greater resilience.

Regarding wood resources, similar results were also identified in [61], which reported that wood uses, especially those related to construction, have shown a tendency for more selective collection, indicating that people tend to select trees with specific diameters, resulting in greater extraction pressure in certain phases of the life cycle of plant individuals. According to [32], the demand for species of this size for timber purposes is concerning, as high exploitation can damage vegetation and cause serious impacts on ecosystems. The quality and yield in the timber extraction process depend on the shape of the stem, without flattening or curvature. These woods from young and larger trees would have greater acceptance in the market due to their straight or less curved shape [62]. In this sense, although the consequences of extractive practices have not been extensively evaluated, the reported usage patterns of *T. glabrescens*, combined with local demand, especially for timber and medicinal purposes, suggest a potential risk of overexploitation. While these observations are based on a specific group of informants, the consistency between their reports and the structural gaps observed in the field plots (i.e., the scarcity of larger individuals) warrants attention. Therefore, despite the limitations in sample representativeness, the data gathered here provide preliminary evidence that can contribute to the management and sustainable use of the species.

### Gender affects the collection pattern

Our findings revealed a significant association in the use citations between genders, showing that men tend to mention a wider variety of uses. There was also a significant association between use categories and gender, with men having higher citations in all categories. This difference can also be attributed to divisions in the collection between genders, in which it is common for men to be responsible for searching for forest resources, highlighting the influence of social divisions of gender within a community, which can directly impact the relationships of use and management of local vegetation [63]. Oliveira et al. [32] investigated the uses of *Vellozia aff. sincorana*, mainly used as fuel to ignite fires in wood stoves, and found that men had greater knowledge about the species compared to women due to the historical use of these plants in rudimentary mining activities in the past, which were performed almost exclusively by men.

Similar results were reported by [63], which have indicated that men had broader knowledge about plants for construction categories and domestic goods, encompassing aspects associated with utensils, compared to women in the community who showed greater knowledge related to the medicinal category [64]. observed that in the Fulni-ô indigenous community, located in Northeast Brazil, men have a broader knowledge in terms of the number of medicinal plants compared to women. However, regarding knowledge transmission, the authors noted that sharing among women was more significant, thus demonstrating the influence of gender on the local knowledge system.

Contrasting results were found by [65], who, when seeking to define the knowledge and use patterns of medicinal plants in traditional communities, did not observe statistically significant relationships between richness of knowledge and the informants’ gender [66], when analyzing knowledge of plant uses at different scales, did not detect significant differences globally, with men and women showing similar knowledge at the national and continental levels, although some significant differences were observed in specific contexts. However, the authors also noted a lack of significant differences in gender knowledge, which reinforces the framework of Howard [12], pointing out that gender is not merely a statistical datum but a fundamental analytical category for understanding the motivations and distinct exploitation pressures on plant resources. While gender is a significant factor in shaping the knowledge and use patterns of *T*. *glabrescens*, it is likely part of a broader set of variables, such as resource availability and competition with other regional species, which were not the primary focus of this study. Nevertheless, identifying gendered differences in collection patterns is a fundamental step for developing inclusive management plans. These distinctions allow for the identification of specific actors - such as those focused on timber (man) versus medicinal uses (woman) - ensuring that sustainability strategies address the diverse harvesting pressures exerted by different groups within the community. These studies may indicate that broader contextual and cultural variables need to be considered when interpreting gender differences in plant knowledge. Regarding the community, since *Terminalia glabrescens* is a tree mainly found in the forest, it is possible that the greater knowledge about the species possessed by men is attributed to the time that they frequently dedicate to the collection of resources in the forest, especially in the context of timber use.

### Intense extraction causes population decline

The results of the population status of *Terminalia glabrescens* showed that the species did not fit the reverse J-shaped model, suggesting that collection patterns may impact trees in the intermediate and larger height and diameter classes. The preference for bark harvesting—rather than leaves—is a relevant finding, as interviewees indicated that individuals in the intermediate and upper height classes are those with bark most suitable for extraction. This reported preference coincides with the structural patterns observed in the field plots, where there is a lower density of individuals in these specific size classes. The convergence between the interviewees’ reports and the physical gaps in the population structure suggests that selective harvesting may be a contributing factor to the deviations observed from the typical inverted J-shaped distribution. In addition, through observations made during the field research activity, it was possible to diagnose traces of human impact on the plant, such as cut trunks, bark extraction marks, fences built from the wood, as well as the low number of individuals in the largest diameter classes.

Although our findings do not allow for a direct and long-term assessment of exploitation levels or specific consequences by use category on the species population status, the decrease in trees in these diameter and height classes can mainly be attributed to timber exploitation. This occurs because timber harvesting tends to prioritize larger trees, which are more valued for their size and quality, resulting in selective extraction. The association between the decrease of individuals in these classes and exploitation activity indicates possible consequences for the ecological balance of the species in the studied region. This ecological scenario must be interpreted alongside the socioeconomic data; although timber use was not always explicitly reported by all participants, likely due to legal restrictions associated with wood extraction, it was indirectly evidenced. Such evidence emerged both during interviews, through cautious statements like *“it can be used to make fences*,* but I do not use it”*, and through the direct field observations of harvested individuals previously described. Thus, the differentiation between knowledge and use varied according to the use category. While medicinal and utilitarian uses were openly reported as practical experiences, timber-related uses (construction and fuel) were frequently described as theoretical knowledge.

The finding that timber use categories are more related to the decline in plant species populations than other categories is an observation found in the study by [67], where the correlation between construction and fuelwood categories and the perceived plant population decline revealed that these categories are responsible for the greatest pressure on the use of medicinal plants.

In this context, wood collection for fuel use is a common practice in rural communities, and usage patterns can have significant impacts on plant structure. The effects of fuelwood collection were investigated by [68], and the removal of canopy trees for fuelwood induced depressed seed dispersal processes and affected tree recruitment success. Additionally, the collection practice can often focus on a specific group of species [51]. found that wood extraction led to greater uniformity in the plant community, negatively affecting overall density. The authors reported that dominant species were not affected by wood collection, while species most used for fence construction are potentially threatened.

In the studied community, located within an ecotonal ecosystem, *T*. *glabrescens* does not represent the only source of forest resources. Other woody species widely distributed in the region [69], such as *Parkia pendula* (Willd.) Benth. ex Walp., *Hymenaea stigonocarpa* Mart. ex Hayne, and *Qualea grandiflora* Mart., are also used for similar purposes, particularly for timber and rural construction, as indicated by ongoing studies conducted by our research group in this community, as observed in another ethnobotanical research [70]. However, the selective pressure observed in our results reflects a broader socioecological context in which *T. glabrescens* ranks among the most frequently used species, likely due to technological wood properties such as strength and durability. Therefore, the impact recorded in its population structure should be interpreted as part of a multispecies management system, in which the high demand for specific use categories (e.g., timber and construction) concentrates harvesting pressure on a limited set of key species within the local ecosystem.

A previous study investigating the impact on *Copaifera langsdorffii* and *Terminalia glabrescens* showed that plant height and stem thickness did not influence bark regeneration in the latter, which exhibited a higher regeneration rate than the other species [17]. According to those authors, bark extraction from this species should preferentially be performed on large, thicker individuals. This corroborates the local preference for higher height classes identified in our interviews, indicating that, over time, people have perceived that selecting larger specimens is the best strategy for bark harvesting when the focus is medicinal. However, when the resource is harvested for timber, the impact is greater, given the participants’ preference for its wood as well.

A limitation of the present study is the absence of direct assessments regarding regeneration dynamics, as well as the difficulty in obtaining interview data directly related to timber use that could be correlated with field observations, such as bark stripping, harvested individuals, and the scarcity of individuals in larger diameter classes, due to the legal implications involved. Although the lack of an undisturbed area, with a population without signs of extraction to serve as a control for comparison with the sampled plots, also constitutes a limitation, processes such as low recruitment and reduced growth rates may occur before more evident structural changes manifest. Nevertheless, ethnobotanical information on use categories, harvested parts, and collection patterns, combined with the observed population structure, provides consistent indirect evidence that intense and selective extraction, especially when targeting larger individuals for timber and energy purposes, may compromise population stability.

## Conclusion

*Terminalia glabrescens* constitutes an important local plant resource for the studied community, particularly for timber and medicinal purposes. Our findings demonstrate that knowledge and exploitation patterns are gendered: men focus primarily on timber extraction, while women are the leading holders of knowledge regarding medicinal bark. This distinction is crucial for management plans, as it identifies different stakeholders and specific harvesting pressures. Furthermore, the local preference for harvesting bark from larger individuals aligns with previous biological evidence indicating that plant size does not negatively affect bark regeneration in this species. This suggests that the community has developed an adaptive strategy for medicinal use that favors specimens with higher recovery rates. However, the integration of specialized ethnobotanical reports with population structure analysis indicates that when the harvesting focus shifts to timber, the impact is significantly higher. Thus, the observed structural gaps in the forest plots are likely driven by timber demand rather than medicinal use, highlighting the need for conservation strategies that address the sustainability of wood extraction in this Cerrado-Caatinga transition zone.

## Data Availability

The data that support the findings of this study are held by the Federal University of Piauí (UFPI) under the supervision of Dr. José Ribamar Sousa Júnior and are available from the authors upon reasonable request.
